# Shift of EMT gradient in 3D spheroid MSCs for activation of mesenchymal niche function

**DOI:** 10.1038/s41598-017-07049-3

**Published:** 2017-07-31

**Authors:** Sohee Jeon, Ho-Sun Lee, Ga-Young Lee, Gyeongsin Park, Tae-Min Kim, Jihye Shin, Cheolju Lee, Il-Hoan Oh

**Affiliations:** 1 Catholic High-Performance Cell Therapy Center, The Catholic University of Korea, College of Medicine, Seoul, 137-701 Korea; 2 Department of Medical Lifescience, The Catholic University of Korea, College of Medicine, Seoul, 137-701 Korea; 3 Department of Pathology, Seoul St. Mary’s Hospital, The Catholic University of Korea, College of Medicine, Seoul, 137-701 Korea; 4 Department of Medical Bioinformatics, The Catholic University of Korea, College of Medicine, Seoul, 137-701 Korea; 5 Center for Theragnosis, Biomedical Research Institute, Korea Institute of Science and Technology, Seoul, 02792 Korea

## Abstract

Despite the wide use of mesenchymal stromal cells (MSCs) for paracrine support in clinical trials, their variable and heterogeneous supporting activity pose major challenges. While three-dimensional (3D) MSC cultures are emerging as alternative approaches, key changes in cellular characteristics during 3D-spheroid formation remain unclear. Here, we show that MSCs in 3D spheroids undergo further progression towards the epithelial-mesenchymal transition (EMT), driven by upregulation of EMT-promoting microRNAs and suppression of EMT-inhibitory miRNAs. The shift of EMT in MSCs is associated with widespread histone modifications mimicking the epigenetic reprogramming towards enhanced chromatin dynamics and stem cell-like properties, but without changes in their surface phenotype. Notably, these molecular shifts towards EMT in 3D MSCs caused enhanced stem cell niche activity, resulting in higher stimulation of hematopoietic progenitor self-renewal and cancer stem cell metastasis. Moreover, miRNA-mediated induction of EMT in 2D MSCs were sufficient to mimic the enhanced niche activity of 3D spheroid MSCs. Thus, the molecular hierarchy in the EMT gradient among phenotypically indistinguishable MSCs revealed the previously unrecognized functional parameters in MSCs, and the EMT-enhanced “naïve” mesenchymal state represents an ‘activated mesenchymal niche’ in 3D spheroid MSCs.

## Introduction

Mesenchymal stromal cells (MSCs) are nonhematopoietic adherent cell populations derived from various organs, including the bone marrow (BM), adipose tissue, and placental tissue. Studies have shown that the primary mode of action of MSCs is establishment of a regenerative microenvironment in response to tissue injury, thereby stimulating the regeneration of endogenous stem cells, such as hematopoietic stem cells (HSCs), neuronal stem cells, or other tissue-specific stem cells^1, 2^.

During development, MSCs are derived from the neural crest through the epithelial-mesenchymal transition (EMT) to form the hematopoietic niche in the BM, or from mesenchymal sclerotome that contribute to the osteochondral differentiation^3^. After birth, MSCs derived from pericytes in various organs^4^ can give rise to self-renewing mesenchymal progenitors, which contribute to stem cell niche in a heterotrophic BM model^5^.

These MSCs are frequently expanded by *in-vitro* culture and these *ex vivo* cultured MSCs have been widely used for cell therapy in a variety of clinical trials. To date, over 320 clinical trials have been registered (www.clinicaltrials.org) for cell therapies aimed at supporting the regeneration of various types of tissue-specific stem cells^1, 2^. However, the outcomes of clinical trials utilizing *ex vivo* expanded MSCs have been characterized by significant variations in outcomes, as exemplified in trials performed to enhance hematopoietic engraftment or immune suppression^6, 7^.

Accordingly, the cellular identities of *ex-vivo* expanded MSCs become controversial. Indeed, it is unclear whether these cultured MSCs can represent the *in vivo* nature of MSCs because they are selectively outgrown from subsets of MSCs during expansion culture^8^. Similarly, studies have shown that MSCs expanded under conventional *in vitro* culture by plastic adherence undergo functional and phenotypic changes during the passage of cultures, creating features that are distinct from those of *in vivo* isolated MSCs^9^. In addition, our recent study showed that the cellular characteristics of MSCs to support stem cells exhibit dynamic changes according to the pathological changes in bone marrow microenvironment or intensity of the canonical Wnt signaling pathways^10, 11^ and can exhibit reversible changes in niche activity by switching of culture conditions^12^, thus pointing possible heterogeneity in the supporting function of MSCs with respect to the culture conditions.

To overcome these limitations, multiple platforms for the *ex vivo* culture of MSCs have been developed that includes three-dimensional (3D) culture or development of polymers with bioactive compounds^13^. In particular, 3D culture platforms have been implicated as an approaches to recapitulate the *in vivo* microenvironment for regeneration of tissues^13^; developmental studies have shown that cells are engaged in the three-dimensional (3D) cellular interactions during embryogenesis and morphogenesis^14^. Moreover, studies on organoid culture showed that pluripotent or tissue-specific stem cells in 3D culture exhibit better tissue-specific function to faithfully recapitulate the *in vivo* tissue development and their regenerative process^15, 16^.

Accordingly, increasing numbers of studies have employed spheroid aggregates of cultured MSCs and found distinction in the pattern of gene expression, increase in cytokine secretion or multi-lineage differentiation compared with those of two-dimensional (2D) cultures of MSCs^17, 18^. However, despite these increasing interests on 3D spheroid MSCs, key changes in the cell fate control of MSCs in 3D cellular interaction and their impact on functional characteristics remains largely unclear.

In the current study, using a polydimethylsiloxane (PDMS)-mediated MSC spheroid formation system, we exploited the key changes in cellular signatures induced by 3D cellular interaction of MSCs. We show that 3D cellular interaction of MSCs drives the cells towards further progression of the EMT process, thus creating molecular heterogeneity in the EMT gradient. These EMT-driven naïve MSCs serve as an activated niche for normal and cancer stem cells, revealing a previously unrecognized parameter for the mesenchymal supporting function of 3D MSCs.

## Results

### MSCs in 3D spheroid culture exhibit enhanced chromatin dynamics for epigenetic plasticity

**Figure 1:**
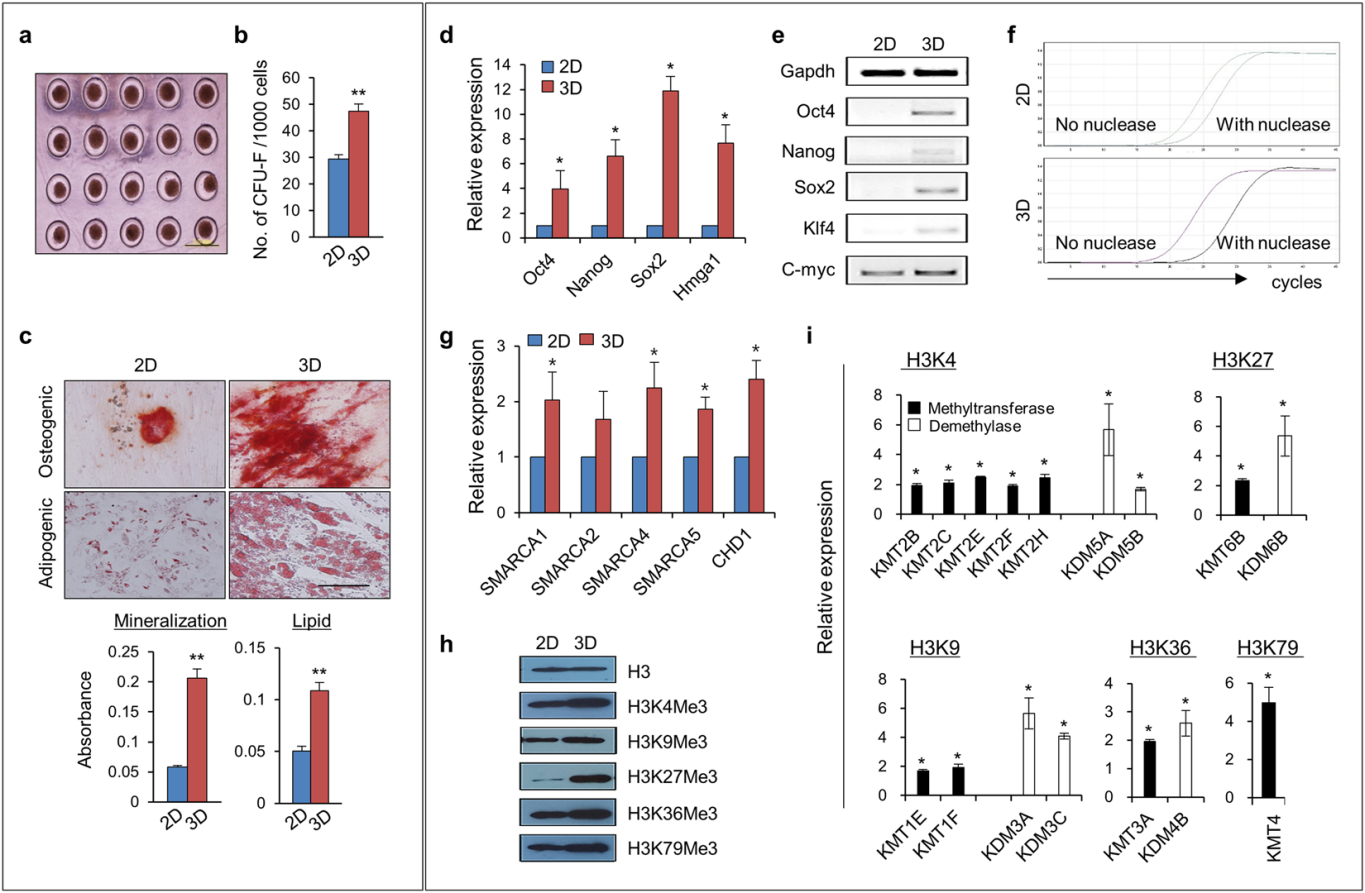
3D spheroid MSCs acquired stem cell-like properties through chromatin dynamics and epigenetic plasticity. (**a**) Light microscopy of adipose-derived MSCs aggregated in PDMS microchips at 24 h after plating (scale bar, 1000 μm). (**b**) Frequency of colony forming unit-fibroblasts (CFU-F) in 2D or 3D MSCs. Shown are the numbers of CFU-F obtained after plating 1 × 10^3^ cells (mean ± SEM, from three experiments; n = 9; **p < 0.01). (**c**) Multi-lineage differentiation of 2D and 3D MSCs. Osteogenic and adipogenic differentiation results were quantified by mineralization (Alizarin red) and lipid droplet deposition (oil red O) after 21 and 14 days of differentiation, respectively. Shown are the representative images (upper) and spectrophotometric quantification (lower) of mineralization and lipid droplet deposition (mean ± SEM, three experiments; n = 6; **p < 0.01). (**d** and **e**) Expression of pluripotency-related genes in 2D or 3D MSCs. Shown are the relative fold changes in expression for the indicated genes in 3D MSCs relative to 2D MSCs, as determined by real-time RQ-PCR with normalization to GAPDH levels (mean ± SEM, three experiments; n = 6; *p < 0.05) (**d**) along with representative images of RT-PCR analysis (**e**). (**f**) Chromatin accessibility to the *Oct4* gene promoter was compared for 2D MSCs (upper) and 3D MSCs (lower) by nuclease protection assays. (**g**) Fold induction of chromatin remodeling genes in 3D MSCs relative to 2D MSCs as determined by qPCR analysis with normalization to *GAPDH* levels (mean ± SEM, three experiments; n = 6; *p < 0.05). (**h**) Western blot analysis of histone methylation marks in total cell lysates (three experiments, n = 3). (**i**) Quantitative analysis of the expression levels of histone methyltransferases and demethylases for each of the indicated histone marks. Shown are the mean fold changes in 3D MSCs relative to 2D MSCs, as determined by qPCR with normalization to *GAPDH* levels (mean ± SEM, three experiments; n = 6; *p < 0.05).

To achieve 3D spheroid formation, MSCs were cultured on PDMS microchips, a platform for 3D culture by ultra-low attachment^19^, and compared with those grown by 2D adherent culture in plastic dishes. The cells grown on PDMS microchips aggregated and formed homogenous spheroids within 24 h (Fig. 1a). MSCs within the spheroids were smaller in size than MSCs in 2D culture (Supplementary Fig. S1a,b) and became more quiescent in cell cycling, as determined by Ki67 staining for detection of proliferation (Supplementary Fig. S1c,d). However, MSCs within 3D spheroids exhibited significantly higher colony forming unit-fibroblasts (CFU-F), along with enhanced osteogenic and adipogenic differentiation potential compared with MSCs grown under 2D adherent culture conditions (Fig. 1b,c), similar to the observations from other 3D platform of MSCs^17, 18^. Notably, 3D spheroid MSCs exhibited higher expression levels of pluripotency-related genes, such as *Oct4*, *Nanog*, and *Sox2* ^20^, and stemness-related genes, such as *HMGA1* ^21^ (Fig. 1d,e). The induction of pluripotent genes in 3D-spheroid MSCs was associated with increased nuclease accessibility to the *Oct4* promoter region (Fig. 1f), along with induction of chromatin remodeling genes, such as *SMARCAs* and *CHD1* (Fig. 1g), thus indicating that MSCs in 3D spheroid culture exhibited stem cell-like properties associated with dynamic remodeling of chromatins.

To further examine the dynamic chromatin remodeling of 3D MSCs, we next examined their epigenetic modifications by comparing chromatin marks and histone-modifying enzymes in MSCs cultured under 2D or 3D conditions. As shown in Fig. 1h, active chromatin marks, such as tri-methylation of H3K4, H3K36, and H3K79^22^, were increased, along with a marked increase in H3K27, accompanied by increased expression levels of each specific histone methyltransferase, i.e., KMT2B, KMT2C, KMT2E, KMT2F, and KMT2H (for H3K4 methylation); KMT1E and KMT1F (for H3K9 methylation); KMT6B (for H3K27 methylation); KMT3A (for H3K36 methylation); and KMT4 (for H3K79 methylation) (Fig. 1i). However, the spheroid MSCs also exhibited simultaneous increases in the expression of counteracting, specific histone demethylases, such as KDM5A and KDM5B (for H3K4 demethylation), KDM3A and KDM3C (for H3K9 demethylation), KDM6B (for H3K27 demethylation), and KDM4B (for H3K36 demethylation) (Fig. 1i), thus indicating that 3D MSCs exhibited increased turnover of histone methylation/demethylation for more dynamic modifications of chromatins. Taken together, these findings indicated that spheroid MSCs in 3D cellular interaction acquired stem cell-like properties characterized by dynamic chromatin remodeling and increased turnover in histone modification, thereby recapitulating the epigenetic plasticity of primitive state cells^23, 24^.

### MSCs in spheroid culture were further driven toward the EMT

To further characterize the cellular changes occurring in 3D spheroid MSCs, we next compared the microRNA (miRNA) expression profiles of 3D and 2D MSCs, based on the key regulatory roles of miRNA in cell fate control^25^. In total, 166 miRNAs were upregulated (log_2_ fold change ≥2), whereas 175 were downregulated (log_2_ fold change ≤−2) in 3D spheroid MSCs (Supplementary Fig. S2a). The top 50 upregulated and top 50 downregulated miRNAs in 3D culture are summarized in Supplementary Fig. S2b.

**Figure 2:**
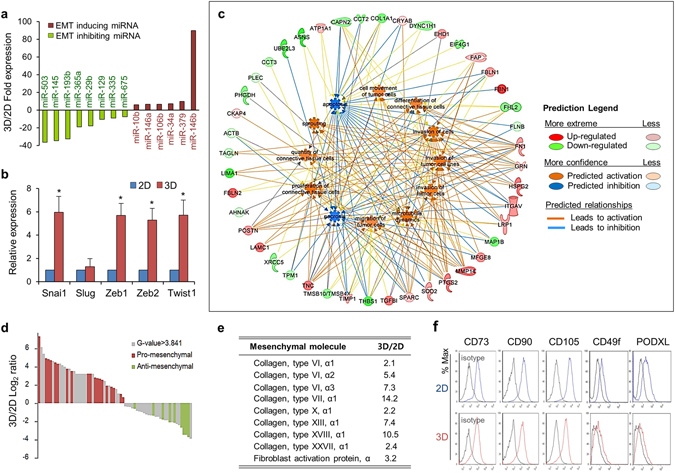
3D spheroid MSCs were further driven towards the EMT. (**a**) Profiles of miRNAs regulating the EMT process among the top ranked 50 miRNAs exhibiting differential expression between 3D and 2D MSCs. The red bars indicate EMT-promoting miRNAs, and green bars indicate EMT-inhibiting miRNAs. (**b**) Induction of EMT-promoting molecules in 3D MSCs in comparison with those in 2D MSCs. Relative fold changes in the expression levels of the indicated genes were determined by qPCR with normalization to *GAPDH* levels (mean ± SEM, three experiments; n = 6; *p < 0.05). (**c** and **d**) Mesenchymal shift in the EMT gradient of 3D MSCs based on proteomic analysis. The non-redundant proteins identified in liquid chromatography-mass spectrophotometry for two independent sets of 3D and 2D MSCs were compared. (**c**) Differentially expressed proteins (G value > 3.841) were analyzed for functional annotations related to the EMT, and their networks were generated based on IPA analysis. Up- and downregulated proteins are indicated red and green, respectively. The regulated functions are colored by their predicted activation state: activated (orange) or inhibited (blue). The edges connecting the nodes are colored orange or blue for activation or inhibition of downstream nodes, respectively. (**d**) Shift of proteins towards the mesenchymal state. Proteins with significant differential expression (DEGs; gray) were aligned according to the order of the relative ratio (log_2_ 3D/2D) of expression levels. Among the DEGs, all proteins annotated to increase the EMT were marked with red, and proteins annotated to inhibit the EMT were marked with green. (**e**) Fold changes in mesenchymal molecules in 3D spheroid MSCs relative to expression levels in 2D MSCs. The expression levels of representative mesenchymal molecules in 3D MSCs relative to those in 2D MSCs were analyzed from RNA-seq data. (**f**) The expression of surface markers for MSCs was determined for 2D and 3D cultured MSCs by flow cytometry. The black graph indicates the isotype control, the blue and red graphs indicate 2D or 3D MSCs, respectively.

Interestingly, many miRNAs with prominent expression changes regulate the EMT. For example, *miR-146b*, a key inducer of the EMT^26^, was among the top highly induced miRNAs in 3D spheroid MSCs (89-fold; Fig. 2a). Similarly, the EMT inducers *miR-379* ^27^, *miR-34a* ^28^, *miR-106b* ^29^, *miR-146a* ^30^, and *miR-10b* ^31^ were also highly induced in 3D MSCs. In contrast, many downregulated miRNAs in 3D spheroid MSCs, including *miR-503* ^32^, *miR-145* ^33^, *miR-193b* ^34^, *miR-365a* ^35^, *miR-29b* ^36^, *miR-129* ^37^, *miR-335* ^38^, and *miR-675* ^39^, have been shown to inhibit the EMT (Fig. 2a). Thus, the changes in cell fate reflected by changes in miRNA expression profiles indicated that MSCs in 3D spheroids were further driven towards EMT progression compared with 2D adherent MSCs.

Consistent with these pro-EMT findings in miRNA profiles, the MSCs within 3D spheroids exhibited significantly higher expression levels of genes driving the EMT process, such as *Snai1*, *Twist1*, *ZEB1*, and *ZEB2* ^40, 41^ (Fig. 2b). Moreover, 3D spheroid MSCs exhibited enhanced levels of transforming growth factor (TGF)-β signaling, the master signals driving the EMT^42^ compared with that in 2D MSCs (Supplementary Table S1). Thus, 3D spheroid MSCs were being further driven towards the EMT through this coordinated, multidimensional molecular milieu of factors promoting the EMT.

To further examine this phenomenon at the cellular protein level, we next performed comparative mass spectrophotometry of MSCs under 2D or 3D conditions. From the two independent analyses of each group of MSCs, 1,055 and 1,056 non-redundant proteins were identified. The pathway analysis (IPA) of differentially expressed proteins (G-value > 3.841) identified 10 functional annotations related to the EMT. As shown in Fig. 2c, the proteins activating EMT-related functions (pro-mesenchymal) were mostly upregulated, whereas proteins inhibiting EMT functions (anti-mesenchymal) were downregulated. Interestingly, among these EMT-related proteins, all the proteins upregulated in 3D MSCs (19 proteins) were pro-mesenchymal proteins, whereas all downregulated (10 proteins) in 3D MSCs were anti-mesenchymal proteins, thus creating an EMT gradient at the proteomics level (Fig. 2d). Similarly, 3D MSCs also exhibited an increase in mesenchymal molecules, such as collagen types VI, VII, X, XIII, XVIII, and XXVII and fibroblast activation protein^43^ (Fig. 2e), further supporting increased mesenchymal nature of the cells. However, despite the molecular shifts in spheroid MSCs, their phenotypes were similar to those of MSCs in 2D culture, exhibiting no significant differences in canonical MSC surface markers or those for early passage MSCs during 2D culture such as CD49f or PODXL^44^ (Fig. 2f).

Taken together, these results indicated that further progression of the EMT in spheroid MSCs can induce molecular heterogeneity in mesenchymal nature, thus creating an EMT gradient among the phenotypically indistinguishable MSC population.

### Transcriptome in spheroid MSCs reflects chromatin decondensation and pan-cytokine response

Next, to characterize the changes in MSC spheroids at the transcript level, we examined the transcript profiles of 3D spheroid MSCs in comparison with 2D adherent MSCs by RNA sequencing for 31969 human transcripts. The top 50 and bottom 50 genes with the highest fold changes between MSCs cultured in 3D or 2D conditions were classified as differentially expressed genes (DEGs; Supplementary Table S2). When analyzed by gene set enrichment analysis (GSEA), five and nine gene sets (gene ontology [GO] terms) were found to be significantly enriched (false discovery rate [FDR] <0.2) toward DEGs among the genes with higher or lower expression in spheroid MSCs compared with those in adherent MSCs (Supplementary Table S3).

**Figure 3:**
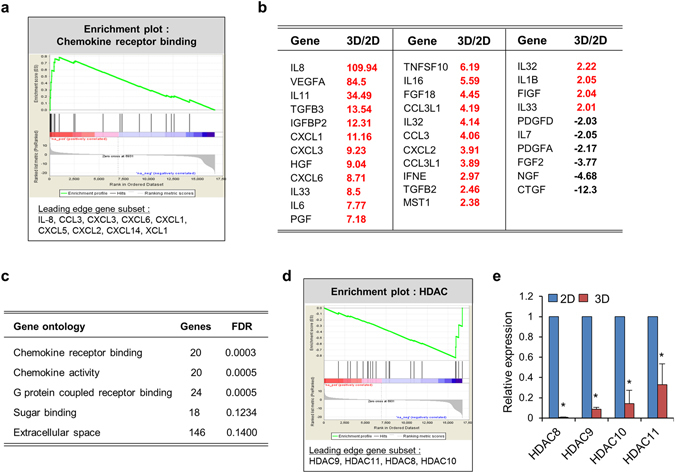
Transcriptomic changes in 3D spheroid MSCs. 3D and 2D MSCs were subjected to whole transcriptome sequencing and relative FPKM was calculated for differential gene expression. Preranked version of GSEA was performed for GO with significant enrichment. (**a**) Enrichment plots of GSEA with significant up-regulation in 3D with leading edge gene subsets indicated. (**b**) Fold changes of transcriptome for cytokine and growth factors in 3D MSC relative to 2D MSC. (**c**) List of significantly (FDR < 0.2) enriched gene sets (GO terms) in 3D MSCs. (**d**) Enrichment plots of GSEA with significant down-regulation in 3D MSCs with leading edge gene subsets indicated. (**e**) RQ-PCR analysis for expression levels of HDACs in 3D MSCs relative to 2D MSCs after normalization with GAPDH (mean ± SEM, from 3 experiments, n = 6; *p < 0.05).

Interestingly, of all the GO terms significantly upregulated in 3D MSCs, those related to cytokine responses, including chemokine receptor binding, chemokine activity, G-protein coupled growth factor signaling, and factors in the secretory extracellular space, comprised most of the transcriptomic changes (Fig. 3a,c).

The individual genes increased in spheroid MSCs compared with those in 2D MSCs encompassed diverse spectrum of cytokine such as interleukin (IL)-8, *IL-11*, and various growth factors, such as *VEGFA*, *TGFB3*, and *IGFBP2* (Fig. 3b). Increased expression of cytokine and growth factor genes was also observed when culture supernatants of 3D or 2D MSCs were compared for secreted proteins using a cytokine array protein blot (Supplementary Fig. S3).

In contrast, the GO terms downregulated in 3D MSCs were not as well characterized. However, among the downregulated GO subsets were histone deacetylase (HDAC) family genes, particularly gene subsets comprised of *HDAC8*, *HDAC9*, *HDAC10*, and *HDAC11* (Fig. 3d,e), further supporting the epigenetic decondensation of chromatin in 3D MSC spheroids.

Together, these findings indicated that chromatin decondensation and increased pan-cytokine production was a characteristic hallmark of functional transcriptomic changes in spheroid MSCs compared with that in adherent MSCs.

### 3D spheroid MSCs represented an activated mesenchymal niche for normal and cancer stem cells

**Figure 4:**
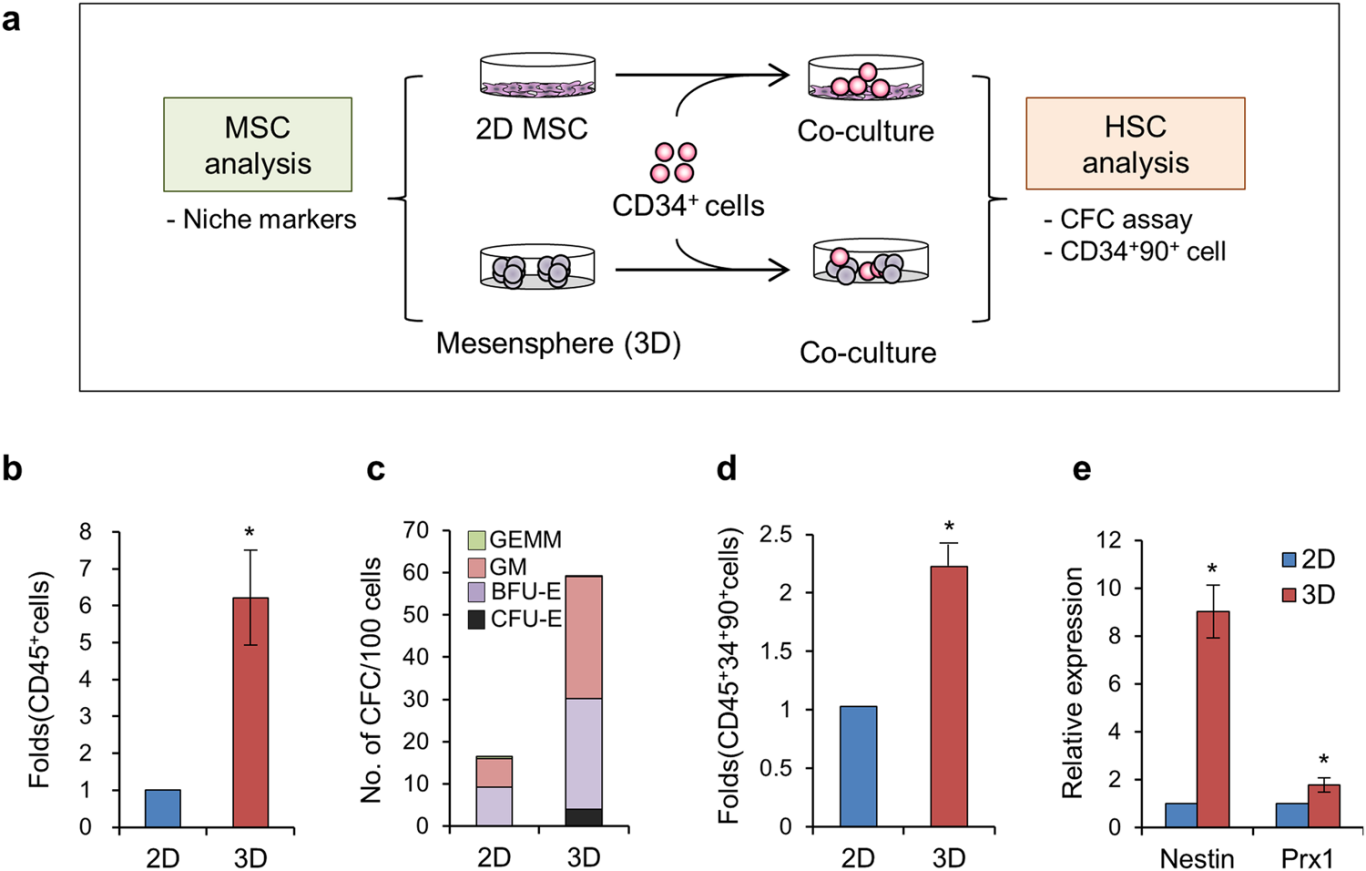
3D spheroid MSCs represented the activated niche for HSCs. (**a**) Schematic illustration of the experimental scheme. (**b**) Expansion of hematopoietic cells after coculture with 2D or 3D MSCs for 5 days. Shown are the mean ± SEM of total CD45^+^ cells (three experiments, n = 3; *p < 0.05). (**c**) Expansion of colony-forming hematopoietic progenitors (CFCs). Mean numbers of CFCs derived from 100 cultured hematopoietic cells (CD45^+^) after coculture are shown with types of each colony for erythroid (CFU-E, BFU-E), granulocyte-macrophage (CFU-GM) and mixed (CFU-GEMM) (three experiments). (**d**) Expansion of undifferentiated hematopoietic progenitor (CD34^+^CD90^+^) cells. Fold changes in coculture with 3D MSCs relative to coculture with 2D MSCs are shown (mean ± SEM, three experiments, n = 3; *p < 0.05). (**e**) Fold changes in expression for niche markers in MSCs determined by RQ-PCR after normalization with GAPDH (mean ± SEM, n = 3; *p < 0.05).

Having observed further progression of the EMT in 3D spheroid MSCs, we next were prompted to determine the functional impact of these differences in the EMT gradient among MSCs, particularly in their stromal niche function to regulate stem cells. Thus, we first examined the ability of spheroid MSCs to support HSCs by coculturing human CD34^+^ hematopoietic progenitor cells with spheroid or adherent MSCs (Fig. 4a). As shown in Fig. 4b and c, CD34^+^ cells exhibited significantly increased proliferation of hematopoietic cells and higher level expansion of clonogenic progenitors when cultured with 3D MSCs than with adherent MSCs. Moreover, CD34^+^ cells cocultured with 3D spheroid MSCs exhibited higher level expansion of hematopoietic subpopulations (CD34^+^CD90^+^) known as long-term repopulating hematopoietic cells^45^ compared with those cultured with 2D MSCs, indicating that HSCs exhibited higher self-renewal capacity (Fig. 4d). Interestingly, examination of mesenchymal niche markers indicated that 3D spheroid MSCs exhibited increased expression of Prx1^46^ or Nestin^47^, the specific markers for *in-vivo* subsets of primitive mesenchymal cells serving as a stem cell niche in the BM (Fig. 4e). Together, these findings indicated that 3D spheroid MSCs underwent cellular changes towards a more activated state of mesenchymal niche to stimulate the regeneration of normal HSCs.

**Figure 5:**
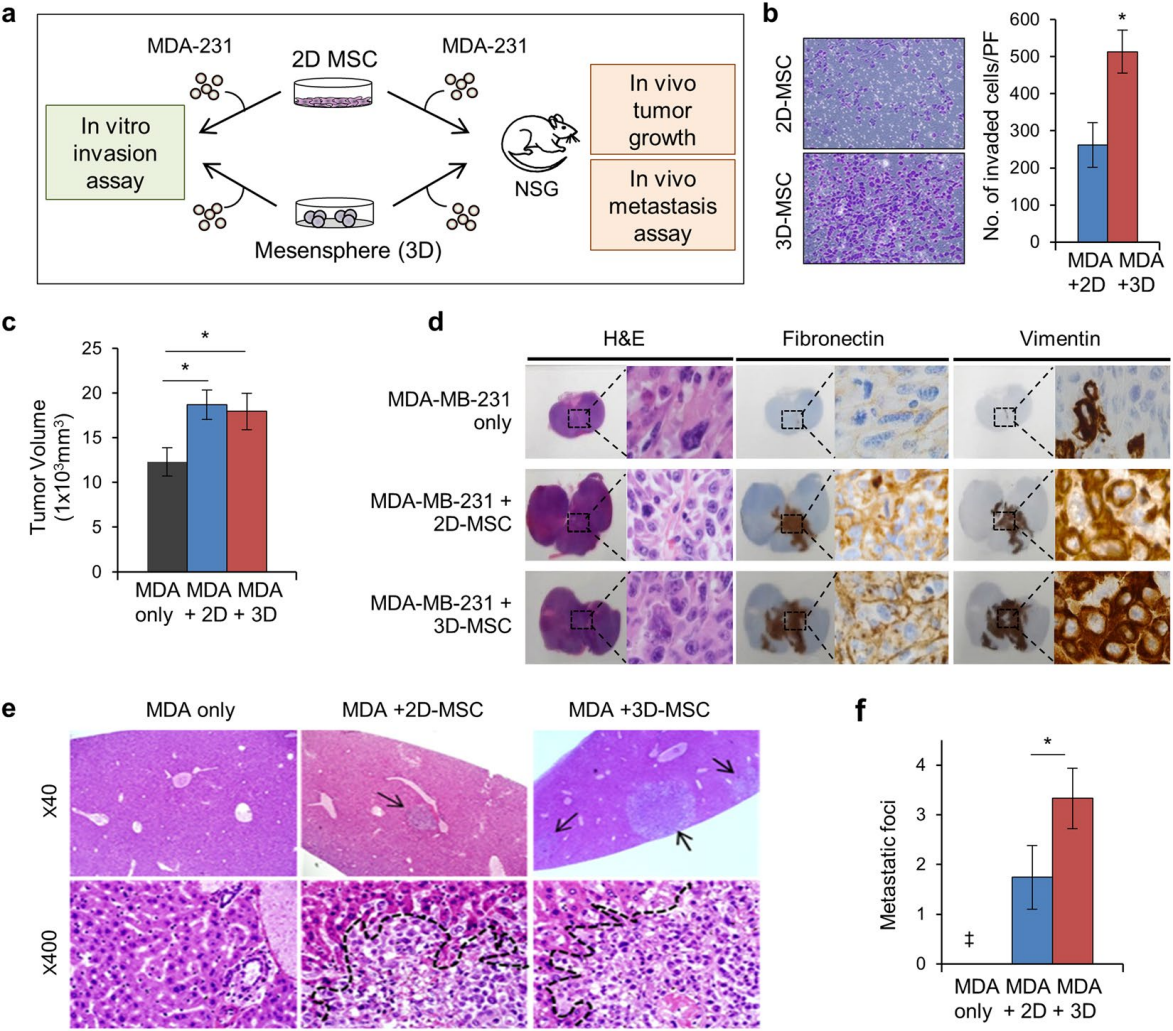
3D spheroid MSCs represented an activated niche for cancer stem cells. (**a**) Schematic illustration of the experimental scheme. (**b**) *In vitro* invasion assay. MDA-MB-231 cells transduced with green fluorescence protein were plated in Matrigel-coated upper chambers of transwell plates with 2D or 3D MSCs. Shown are the representative images after staining with crystal violet and quantitative measurements of invading cancer cells (GFP^+^) for 2D and 3D MSCs (mean ± SEM, three experiments; n = 9; *p < 0.05). (**c**–**f**) Effects of 2D or 3D MSCs on *in vivo* metastasis of tumor xenografts. Breast cancer cells (5 × 10^5^) with or without 2 × 10^5^ MSCs (2D or 3D) were implanted into the mammary fat pads of NOD/SCID-ɤ_c_null mice, and tumor volumes were measured weekly. After 35 days, mice were examined for primary tumors and metastatic lesions. (**c**) Volumes of primary tumors are shown (mean ± SEM; n = 7 each; *, *p* < 0.05). (**d**) Primary tumors were examined by H&E staining and immunohistochemical staining for EMT markers (fibronectin and vimentin). The images were obtained by 1:1 slide scanning in a 1000× power field. Dotted boxes indicate the high-magnification field. Note that large atypical hyperchromatic nuclei denoted fibronectin and vimentin expression in cancer cells. (**e**) Distant metastasis to the liver in each group. Arrows indicate metastatic foci. **(f)** Quantitative analysis of liver metastasis. The numbers of metastatic foci in the liver were counted under a microscope for 20-portal equivalent areas in each xenograft group (mean ± SEM; *p < 0.05; **p < 0.01; ^‡^no metastasis detected).

To examine whether 3D spheroid MSCs can exert analogous effects on other types of stemness, we next compared their effects on stemness in cancer cells using a breast cancer cell model according to previous studies describing the roles of stemness in metastasis of cancer stem cells^48^ (Fig. 5a). First, the effects of these cells on *in vitro* invasion were examined by coculturing MDA-MB-231 breast cancer cells with spheroid or adherent MSCs. As shown in Fig. 5b, breast cancer cells exhibited increased invasion *in vitro* when cocultured with 3D spheroid MSCs compared with that in 2D adherent MSCs, indicating the acquisition of higher metastatic potential^49^. To further test this possibility, we next compared the *in vivo* metastasis of breast cancer cells by implanting the cancer cells into NOD/SCID-ɤ_c_null mice together with each group of MSCs. As shown in Fig. 5c,d, co-implanting either type of MSCs with breast cancer cells increased primary tumor volumes and promoted the progression of the EMT, as shown by the presence of vimentin- or fibronectin-positive areas in primary tumors. However, significant differences were not found between cancer cells implanted with 2D- or 3D-cultured MSCs. In contrast, when distant metastasis of primary tumor cells to the liver was examined, significantly higher numbers of metastatic foci were found in the livers of xenograft mice (Fig. 5e,f). Thus, these findings showed that, while both groups of MSCs could similarly promote *in vivo* tumor growth and EMT progression, 3D-spheroid MSCs caused significant increases in the metastatic phenotype. Together, these results showed that 3D spheroid MSCs served as a functionally activated niche that could stimulate metastasis of cancer stem cells as well as self-renewal of normal HSCs.

### The induction of EMT gradient in 2D MSCs reproduced characteristics of 3D spheroid MSCs

**Figure 6:**
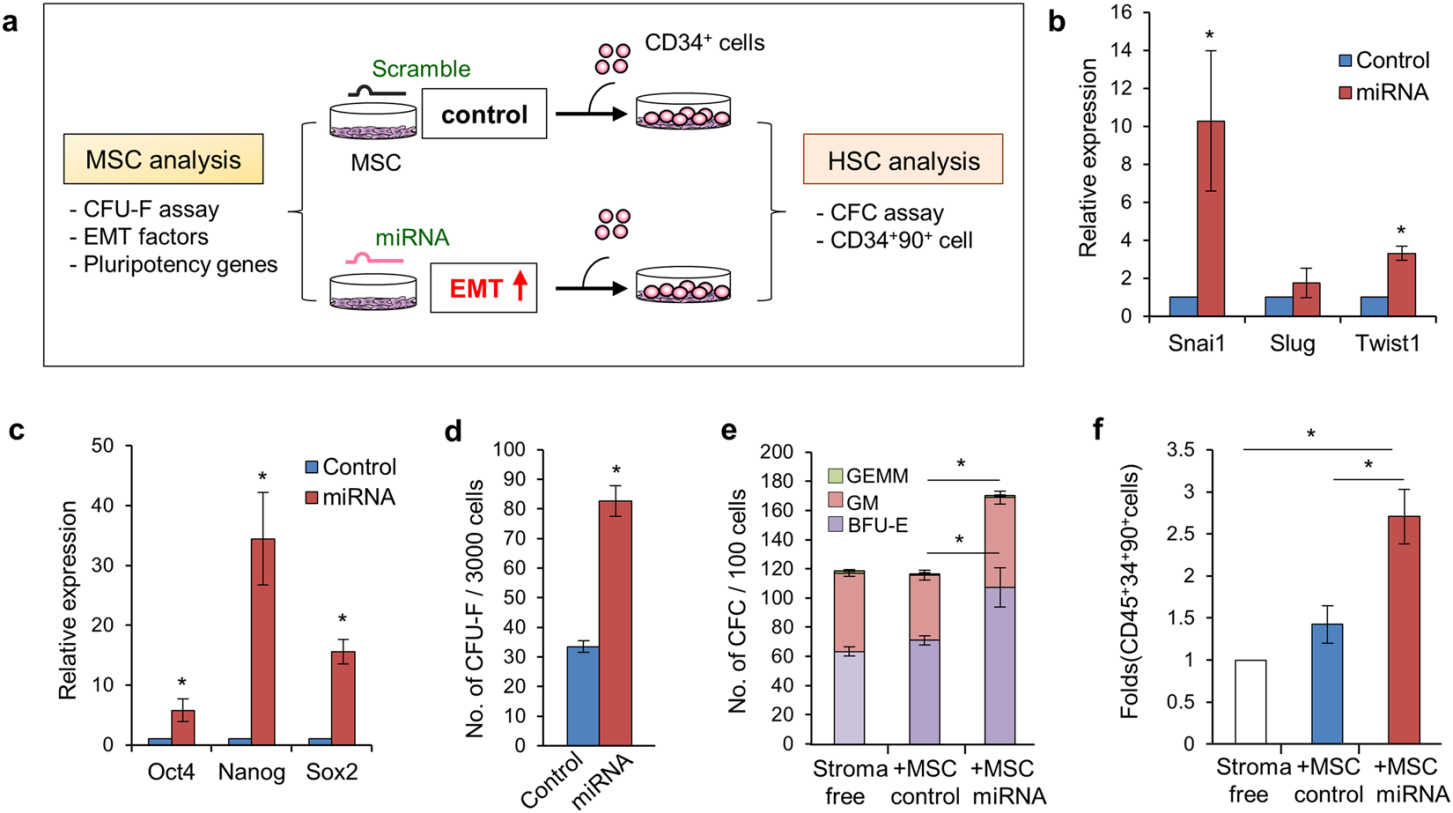
Driving the EMT in 2D-MSCs reproduced the characteristics of 3D spheroid MSCs. (**a**) Schematic of the experimental design. BM-derived MSCs in 2D adherent culture were transfected with miRNA mimetic oligonucleotides for miRNAs promoting the EMT and inhibitory nucleotide for miRNAs suppressing the EMT (miRNA lists and oligonucleotide sequences are shown in the Supplementary Table S5). (**b**–**d**) Effects of EMT-driving miRNAs on 2D MSCs. The expression levels of indicated master EMT-regulatory gene (**b**) or pluripotency gene (**c**) were analyzed in 2D-MSCs transfected with miRNA compared to control (scramble)-transfected MSCs. Relative expression folds are shown with SEM (three experiments). (**d**) Effects of the EMT on mesenchymal progenitors. Shown are the numbers of CFU-F obtained from 3000 MSCs transfected by each miRNA group (three experiments). (**e** and **f**) Effects on the expansion of primitive hematopoietic population. After 3 days of coculture of CD34^+^ cells with MSCs transfected with each group miRNAs, the numbers of CFCs of each lineage (**e**) and primitive repopulating hematopoietic progenitors (CD34^+^90^+^) (**f**) are shown (mean ± SEM; three experiments; *p < 0.05).

Having observed the association of enhanced niche activity with the EMT gradient, we next explored the influence of the EMT gradient on the niche activity of mesenchymal cells (Fig. 6a). To this end, we transfected 2D MSCs with miRNA mimics that could increase the EMT gradient, as in mesenspheres, i.e., those mimicking EMT-promoting miRNAs (*miR-140b*, *miR-379*) and those blocking EMT-inhibiting miRNAs (*miR-503*, *miR-145-5p*, *miR-193b*, *miR-36t5a*, *miR-29b*, and *miR-129*). As expected, the miRNA-transfected 2D MSCs showed a significant induction of EMT-promoting genes, such as *Snail1* and *Twist1*, indicating that cells were pushed towards EMT progression (Fig. 6b). Interestingly, inducing the EMT in 2D MSCs caused a significant induction of the pluripotency-related genes *Oct-4*, *Nanog*, and *Sox2* and a higher frequency of clonogenic mesenchymal progenitors (CFU-F) than control MSCs (Fig. 6c,d). Moreover, when examined for their supportive niche function, significantly higher expansion in hematopoietic progenitors (CFC) and hematopoietic repopulating cells (CD34^+^CD90^+^) was observed in EMT-driven MSCs than in controls (Fig. 6e,f). Together these findings showed that the characteristics of 3D spheroid MSCs are reproduced by shift in EMT gradient, indicating the significance of the EMT gradient for enhanced niche activity in 3D spheroid MSCs.

## Discussion

With an increasing number of clinical trials investigating the use of MSCs for paracrine stimulation of the regenerative process, there have been major interests on the regulatory mechanisms and functional parameters of the niche activity of MSCs. Here, we have exploited the impact of 3D sphere formation on the changes in cellular nature of MSCs, based on recent insights on the 3D cellular interaction as an alternative culture platform for closer recapitulation of *in-vivo* microenvironment^15, 16^.

In this analysis, most prominent changes in their biological functions were related to the support of stem cell properties; 3D MSCs served as an activated niche to support HSC self-renewal to significantly higher levels than 2D adherent MSCs, mimicking the activated stem cell niche in 3D MSCs. Similarly, 3D spheroid MSCs had enhancing effects on the *in vivo* metastatic potential of breast cancer cells, without influencing their primary tumor volumes or EMT. Studies showed that, among the cancer cells undergone EMT, only those acquiring stemness become fully metastatic cancer cells^48^. Therefore, it is conceivable that 3D spheroid MSCs should serve as an activated niche for cancer stem cells as well as normal hematopoietic stem cells.

The mechanisms for enhanced niche functions in 3D spheroid MSCs requires further studies. However, we found that 3D spheroid formation in MSCs induced significantly higher expression levels of Prx1 and Nestin, markers of mesenchymal niche cells that support endogenous HSCs in the BM^46, 47^, indicating their relevance to the acquisition of enhanced niche activity. Moreover, our transcriptome analysis revealed a global increase of gene clusters for cytokine-related response, representing most of the GO for those upregulated in 3D MSCs, thus mimicking the MSCs activated in response to injury signals^1^.

Most strikingly, when examined for molecular changes during 3D sphere formation, the most prominent of which was their molecular shift towards the EMT being driven by coordinated, multidimensional factors, including a shift in the miRNA expression profiles towards EMT progression with marked induction of master regulatory genes for the EMT, such as *Snai1*, *Twist1*, and *ZEB1*,*2*. Furthermore, these shifts in cell fate towards EMT progression were similarly observed in mass spectrophotometry analysis of whole cellular proteins from 2D and 3D MSCs, revealing the shift of EMT gradient in 3D spheroid cells at protein level.

Of note, molecular driving of the EMT in 2D MSCs was sufficient to make the cells mimic the characteristics of 3D MSCs. That is, when the 2D MSCs were further pushed towards EMT progression, these cells exhibited induction of stemness genes and a primitive phenotype including the higher frequency of CFU-F and, most importantly, a significant increase in their niche activity to support HSC self-renewal.

Together, these findings suggest that further shift of the EMT gradient in MSCs towards more mesenchymal nature should be functionally related to stimulatory effects of MSCs as well as their own primitive mesenchymal nature in 3D spheroid MSCs.

Notably, in consistence to our findings, progression of EMT after birth has been implicated in various regenerative cellular response being reactivated as a reactive response to injury. For example, in response to ischemic injury, the epicardium undergoes the EMT to enhance cardiosphere formation, thus serving as niche-like cells^50^. Similarly, regenerating renal progenitor cells were developed in response to renal injury associated with the progression of the EMT in glomerular epithelial cells^51^. In addition, the EMT process has been shown to be a central event in wound healing^52^, and induction of Snail has been shown to play a key role in the repair of the gastrointestinal tract or corneal epithelial cells in response to injury^53, 54^. Further, expression level of Twist1 was correlated to the paracrine secretory activity of MSCs to stimulate proliferation of cell lines^55^. In this sense, the EMT process can be viewed in the light of the intrinsic adaptive reaction of mesenchymal cells in response to tissue injury, facilitating regeneration of neighboring stem cells.

Notably, the shift of the EMT gradient in 3D spheroid MSCs was accompanied by acquisition of a dynamic chromatin configuration and epigenetic plasticity, i.e., enhanced accessibility of nuclease to the *Oct-4* promoter and downregulation of multiple families of HDACs, along with increased expression of the chromatin remodeling complex, indicating less condensed chromatins in these cells, similar to that in the epigenetic changes during reprogramming into pluripotent stem cells^24, 56^.

In particular, upregulation of both histone mark specific methyltransferases and their counteracting demethylase enzymes including those for H3K27Me3 indicated dynamic turnover of histone marks in spheroid MSCs, which was a characteristic epigenetic changes during the reprogramming of cells into pluripotent cells^57, 58^. Thus, these finding suggest that the epigenetic changes induced in 3D MSCs resembles the process of epigenetic reprogramming towards more plastic and stem cell-like status of the chromatin.

At present, the significance of these stem cell-like epigenetic changes in 3D MSCs is not yet clear. However, studies have shown that epigenetic plasticity induced by downregulation of HDACs and upregulation of the chromatin remodeling complex were required for complete progression of the EMT process^41, 59^. Moreover, the process of acquiring stem cell-like properties and the EMT are tightly associated biological processes^48^. where the plastic transition of the cellular nature is enabled by epigenetic changes for chromatin signatures^60^. Therefore, it is conceivable that the chromatin dynamics and epigenetic plasticity shown in 3D spheroid MSCs should be an intrinsic process of facilitating the cell fate transition further shifting the cells in the EMT axis, and thereby exhibiting a heterogeneity in their mesenchymal state among the phenotypically similar MSCs.

**Figure 7:**
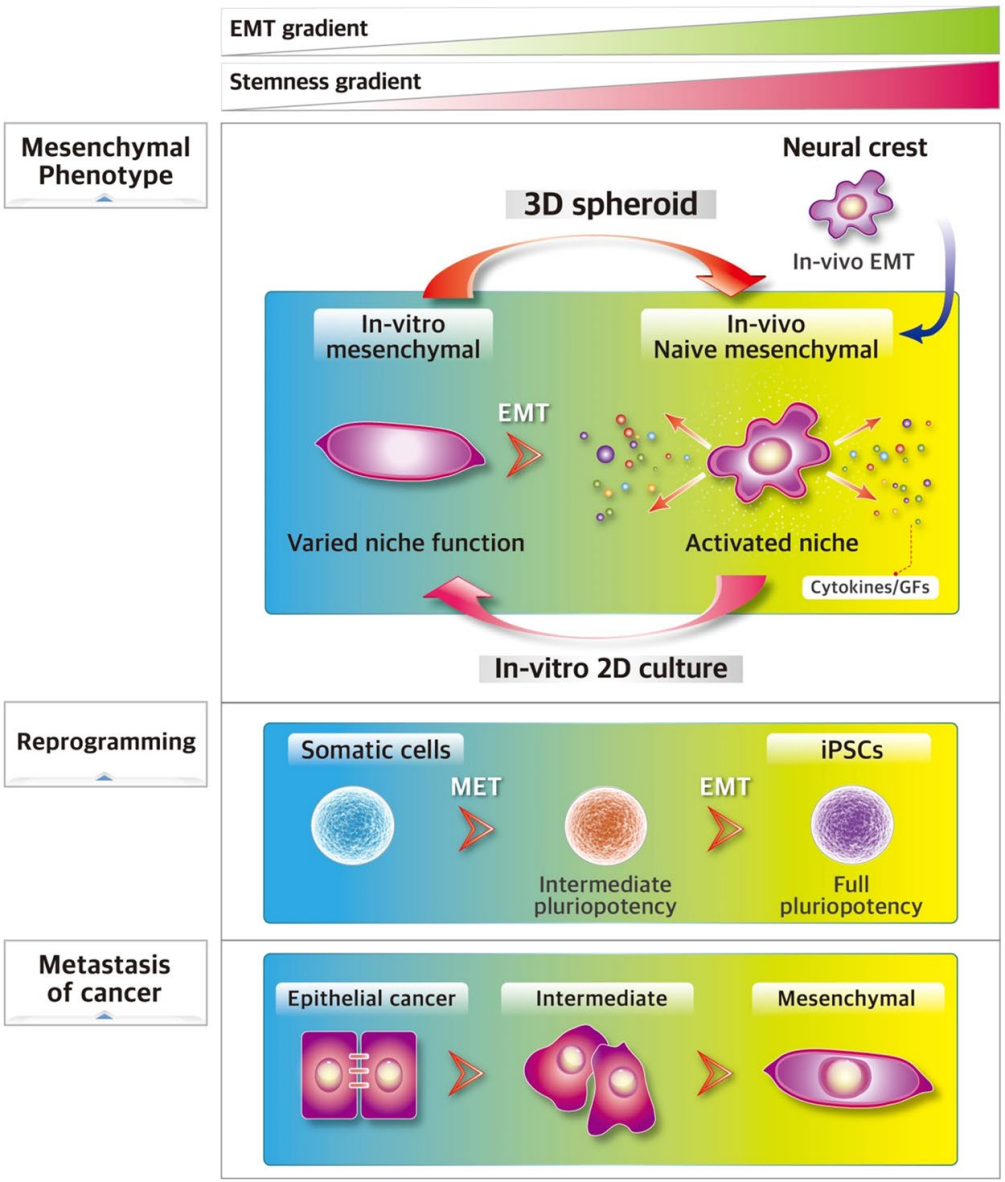
A hypothetical model for the EMT gradient as a functional parameter in cell transitions. MSCs were derived from the neural crest via the EMT, contributing to the *in vivo* mesenchymal niche. During *in vitro* 2D adherent culture, MSCs undergo phenotypic and gene expression changes, resulting in discrepancies from the *in vivo* naïve mesenchymal state and variations in biological niche functions. 3D spheroid culture of MSCs drives the MSCs to a more naïve mesenchymal state, creating molecular heterogeneity in the EMT gradient while preserving the phenotypically redundant mesenchymal phenotype. Models for heterogeneity in the EMT or stemness gradient are also found during cell reprogramming to the pluripotent state; at first, the EMT is downregulated, but is then reactivated during the transition from the intermediate stage to the fully pluripotent stage. Similarly, cancer cells exhibit multiple stages of the EMT/stem cell-like properties, where the hybrid/intermediate stage of the EMT exhibits both epithelial and mesenchymal properties during metastasis. Thus, the heterogeneity in the molecular EMT gradient can be a functional parameter for a broader spectrum of cell transitions.

Of note, while we observed heterogeneities in EMT gradient during 3D sphere formation of MSCs, analogous models of heterogeneity in the EMT gradient have been observed in other biological processes (Fig. 7). For example, proteome analysis of somatic cells undergoing reprogramming to pluripotency has revealed a distinct stage of reprogramming characterized by differences in mesenchymal nature, loss of the EMT at the initial phase, but promotion of the EMT from the intermediate stage to fully pluripotent state^61^. In addition, an analogous heterogeneity in the EMT can be also found in the cancer cell model, i.e., a “partial EMT” stage in the “EMT axis” has been shown to exist, exhibiting hybrid expression of epithelial and mesenchymal characteristics^62^. Taken together, these findings suggest that the heterogeneity in the EMT gradient among phenotypically similar cells may represent a heterogeneity in their functional status among a broad spectrum of biological processes.

In conclusion, our study showed that MSCs in 3D spheroid culture exhibit a previously unrecognized shift in the EMT gradient among the phenotypically similar MSC population, and that this EMT-shifted “naïve” mesenchymal state represents an activated mesenchymal niche’ for normal and cancer stem cells.

## Methods

### Isolation and culture of cells

Human umbilical cord blood, bone marrow or adipose tissues were obtained with informed consent of the all participating donors after approval by the Institutional Review Board of the Catholic University of Korea (KC13MDMS0839, MC12TISI0154). All the study methods were carried out in accordance with the approved guidelines. MSCs were obtained by plating the adipose tissue stromal vesicular fraction or BM mononuclear cells in Dulbecco’s modified Eagle medium (DMEM, Hyclone Laboratories Inc, Logan, UT, USA) with 10% fetal bovine serum (HyClone™ FBS), 2 mM of L-glutamine, and antibiotics (GIBCO, NY, USA). 3D MSC spheroids were generated on PDMS-based concave microchips fabricated using soft lithography techniques^19^. The microchips were coated with 3% (w/v) bovine serum albumin (BSA) to minimize cell attachment. MSCs were aggregated in each microchip for 24 h, and 3D spheroids were suspended in low-adherence dishes (Corning, Lowell, MA, USA) for an additional 24 h.

### Colony-forming unit assays and differentiation

To quantify the frequency of colony-forming cells (CFU-F), MSCs from 2D or 3D culture were resuspended and plated at a density of 1000 cells per 100-mm dish. The frequency of CFU-F was determined by counting after 14 days of incubation after staining with crystal violet (Sigma, St. Louis, MO, USA), as previously described^63^. For osteogenic or adipogenic differentiation, equivalent numbers of MSCs from 2D or 3D culture were seeded (35,000 cells per well) and cultured until both group reached confluency, then switched to differentiation-inducing medium. Mineralization was then quantified using Alizarin Red S, and lipid droplets were quantified by Oil red O, as previously described^10^.

### Protein blots for cytokines

For quantification of cytokine secretion from MSCs, cells were rinsed twice with serum-free DMEM, incubated in serum-free DMEM for 12 h, and analyzed by using a custom-designed membrane array for cytokines (RayBiotech Inc., Norcross, GA, USA) according to the manufacturer’s instructions. Briefly, membranes were blocked and incubated with culture supernatants (1 mL) for 2 h at room temperature. After incubation, the membranes were washed five times with washing buffer I and II for 5 min each, followed by incubation with biotin-conjugated anti-cytokine primary antibodies for 2 h at room temperature. The membranes were then washed and incubated with horseradish peroxidase (HRP)-conjugated streptavidin for 2 h at room temperature and visualized using a chemiluminescence substrate. The densities of individual spots on the array were measured using Image J software (National Institutes of Health; Bethesda, MD, USA). The positive controls on membrane were used to normalize the signal intensities of individual cytokines.

### Real-time quantitative polymerase chain reaction (qPCR) and western blotting

Total RNA, including miRNAs, from 2D- and 3D-cultured MSCs were extracted using TRIzol reagent according to the manufacturer’s protocol (Invitrogen, Paisley, UK). qPCR was performed in triplicate using SYBR Premix Ex Taq II (TaKaRa, Shiga, Japan) and specific primers (Supplementary Table S4), as described^10^. Normalization and fold changes were calculated using the ΔΔCt method. For western blot analysis, MSC lysates were subjected to sodium dodecyl sulfate polyacrylamide gel electrophoresis (SDS-PAGE), transferred to membranes, and incubated with primary antibodies against each specific histone modification. The membranes were then incubated with horseradish peroxidase-conjugated secondary antibodies, and proteins were visualized using a chemiluminescence substrate (Thermo Scientific, IL, USA). All primary antibodies against modified histones were purchased from Abcam (UK).

### *Ex-vivo* culture of hematopoietic cells

Human CD34^+^ cells were purified from umbilical cord blood using a Dynabeads 34 positive isolation kit (Miltenyl Biotec, Bergisch Gladbach, Germany). For coculture, MSCs from 2D or 3D culture were irradiated (1,500 cGy) 24 h before and subsequently cocultured with purified CD34^+^ cells for 5 days in long-term culture medium (H5100; STEMCELL Technologies, Vancouver, Canada) in the presence of a cytokine mixture (100 ng/mL human SCF; 100 ng/mL human Flt3L; and 20 ng/mL human IL-3, IL-6, and G-CSF; ProSpec-Tany TechnoGene Ltd., Rehovot, Israel). For phenotypic analysis of *ex vivo* expanded hematopoietic cells, cocultured cells were stained with antibodies against CD45 (BD Pharmingen, San Jose, CA), CD34 (BD Pharmingen), and CD90 (BD Pharmingen) and then analyzed by flow cytometry after gating the CD45^+^ population, as previously described^63^. Colony-forming assays were performed by plating the cocultured cells in semisolid medium (H4230; STEMCELL Technologies) for 14 days. The number and type of colony, such as colony-forming unit erythroid (CFU-E), burst-forming unit erythroid (BFU-E), colony-forming unit granulocyte–macrophage (CFU-GM), and colony-forming unit granulocyte–erythroid–macrophage–megakaryocyte (CFU-GEMM), were determined by microscopic examination of the colony.

### Invasion assay

The human breast cancer cell line MDA-MB-231 was purchased from ATCC (Manassas, VA, USA). For invasion analysis, 5 × 10^4^ of GFP-transduced MDA-MB-231 cells were seeded in the upper chambers of plates (with 8-μm pores; Corning) coated with growth factor-reduced Matrigel (BD) in serum-free medium. The lower chamber was filled with medium containing 10% FBS as a chemoattractant. After 24 h of incubation, migrated cells on the lower side of membrane were examined for GFP, fixed, and stained with 1% crystal violet. Total cell numbers were counted and quantified with Image J software.

### Whole-transcriptome and miRNA sequencing

MSCs were subjected to whole-transcriptome sequencing (RNA-seq). The paired sequencing reads were aligned onto the reference human genome sequences (hg19) using TopHat. The estimation of gene expression (fragments per kilobase of exon per million fragments mapped [FPKM]) was performed using CuffLink software. For functional interpretation, a preranked dataset of GSEA using the log_2_ of the fold change (FPKM3D/FPKM2D) was analyzed. The GO gene sets that showed significant enrichment (nominal *P* < 0.05) were selected with leading edge genes.

For miRNA sequencing, the extracted total RNAs were resolved on a denatured 15% polyacrylamide gel. Gel fragments of 18–26 nucleotides were excised, and small RNAs were eluted overnight with 0.5 M NaCl at 4 °C and precipitated by ethanol. A small RNA sequencing library was generated with 1 μg of RNA using a TruSeq Small RNA Sample Prep Kit version 2 (Illumina, San Diego, CA, USA). Illumina HCS (version 1.4.8), RTA (version 1.12.4.2), and CASAVA (version 1.8.2) software were used for base-calling and the generation of raw, de-multiplexed sequencing data in FASTQ format.

### Transfection of miRNA

The miRNA mimics, miRNA inhibitors (AccuTarget Human miRNA) were purchased from Bioneer Corp. (Daejeon, Korea). BM-MSCs were cultured in Opti-MEM (Life Technologies, Carlsbad, CA, USA) and transfected with 100 nM miRNA mimetics, miRNA inhibitors using Lipofectamine 2000 (Life Technologies). Scrambled miRNA was used as negative control. The oligonucleotide sequences are shown in the Supplementary Table S5.

### Chromatin accessibility test

Accessibility of chromatin to the *Oct-4* gene promoter in MSCs was assessed by nuclease protection assays^64^ using a chromatin accessibility assay kit according to the manufacturer’s instructions (Epigentek, Farmingdale, NY, USA). Briefly, cells were incubated in EpiQuik chromatin buffer with or without nuclease, and genomic DNA was extracted. Chromatin accessibility was assessed by qPCR and analyzed using the analysis tools in the kit. The β hemoglobin (*HBB*) gene was used as the inaccessible reference for measuring relative chromatin accessibility.

### Liquid Chromatography – Tandem Mass Spectrometry (LC-MS/MS)

The tryptic digests of the cellular proteins were analyzed by nano-flow LC-MS/MS using an LTQ XL-Orbitrap mass spectrometer (Thermo Scientific). Survey full-scan MS spectra (300–2000 m/z) were acquired from the Orbitrap with one microscan and a resolution of 100,000, allowing preview mode for precursor selection and charge-state determination. MS/MS spectra of the 10 most intense ions from the preview survey scan were acquired concurrently from the ion-trap.

The MS/MS spectra were searched against the Swissprot human database (released in 2016.01) using SEQUEST in Proteome Discoverer 1.4 (Thermo Fisher Scientific, version 1.4.0.288). The relative abundance of identified proteins was estimated on the basis of peptide-spectrum matches. The G-value was calculated to determine the differentially expressed proteins (DEPs), and proteins with a value of more than 3.841 were considered significant, with *P* < 0.05 according to the χ2-distribution^65^. Gene ontology analysis of the DEPs was carried out using Ingenuity Pathway Analysis (IPA; Ingenuity system, http://www.ingenuity.com).

### Mouse xenograft assay

Animal experiments were approved by the Catholic University of Korea Animal Care and Use Committee. All experiments were performed in accordance with the institutional animal care and use committee guidelines. NOD/SCID-ɤ_c_null mice (female) were purchased from Jackson Laboratory (Bar Harbor, ME, USA). A total of 5 × 10^5^ MDA-MB-231 cells were subcutaneously implanted in the mammary fat pad with or without 2 × 10^5^ MSCs cultured under 2D or 3D conditions. All mice were sacrificed on day 35. Primary tumors were examined for volume and weight. Primary tumors and livers were fixed, and sections of fixed tissues were immunohistochemically analyzed. The metastatic lesions in the liver were examined by microscopy. The number of metastatic nodules in the liver was counted per 20-portal area fields.

### Data availability statement

“The mass spectrometry proteomics data have been deposited to the ProteomeXchange Consortium via the PRIDE [1] partner repository with the dataset identifier PXD006653”. The datasets generated during smRNA-seq analysis are available in Sequence Read Archive (SRA) repository in NCBI (https://www.ncbi.nlm.nih.gov/sra) with accession number (SRP108655). Additional transcriptomic analysis for all genes are attached as supplemental information of current manuscript.

## Electronic supplementary material

Supplemental information

Supplementary Dataset 1
