# Supplementary material for: Shift of EMT gradient in 3D spheroid MSCs for activation of mesenchymal niche function
Source: Sci Rep. 2017 Jul 31;7:6859. doi: 10.1038/s41598-017-07049-3 (PMC5537359; doi:10.1038/s41598-017-07049-3)
Supplement: Supplementary file 1 — Supplemental information [file 41598_2017_7049_MOESM1_ESM.pdf]

Scientific Reports
Supplemental Information

Shift of EMT gradient in 3D spheroid MSCs for activation of mesenchymal niche function

Sohee Jeon[1,2], Ho-Sun Lee[1,2], Ga-Young Lee[1,2], Gyeongsin Park[3], Tae-Min Kim[4], Jihye Shin[5], Cheolju Lee[5], Il-Hoan Oh[1,2,*]

SUPPLEMENTAL FIGURES

Figure S1

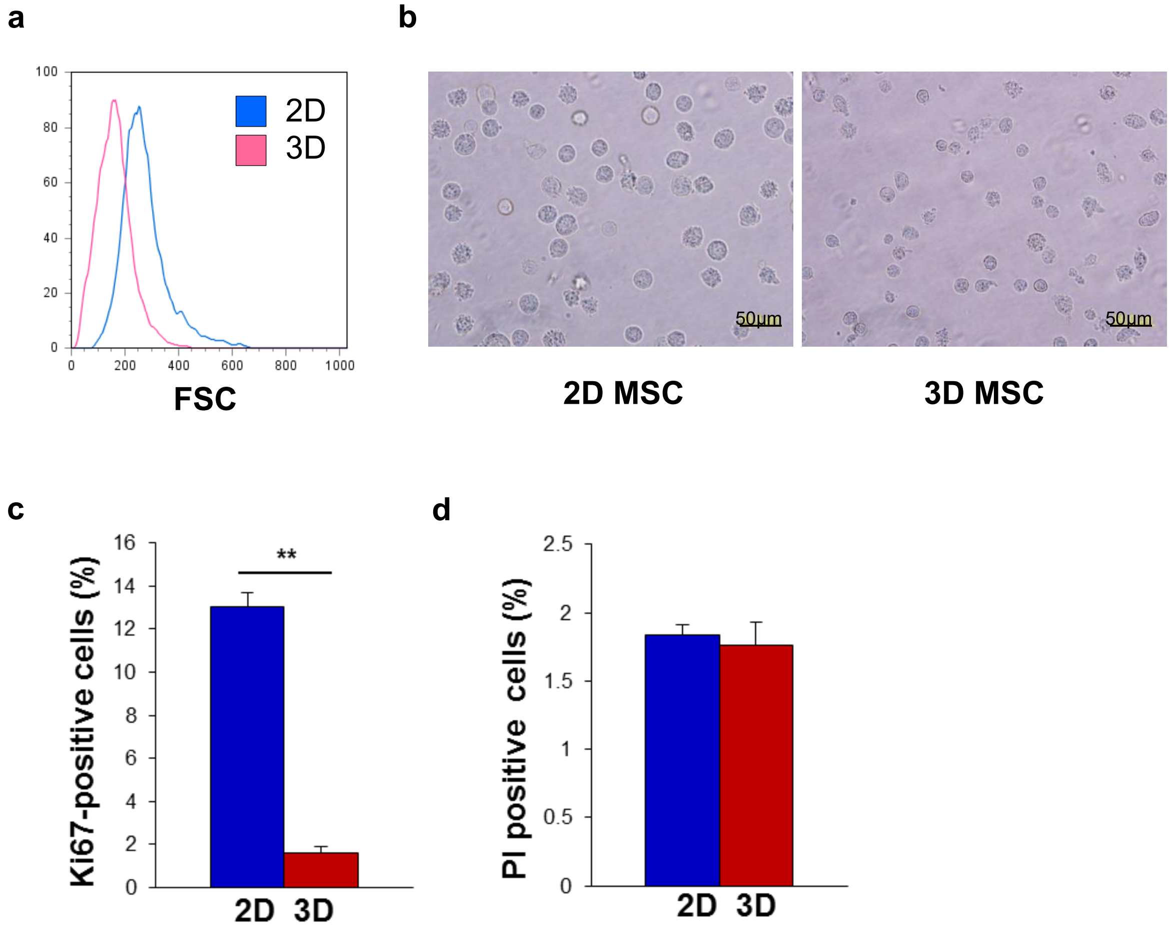

Figure S1. Decreased cell size and proliferative activity of MSCs in 3D culture. (a) Comparisons of cell size between 2D or 3D MSCs analyzed by forward scatter (FSC-A) in flow cytometry. Shown are the representative plots from at least 3 independent experiments. **(b)** Single cell suspension of 2D and 3D MSCs were photographed under light microscopy (bar = 50μm). **(c)** Proliferative activity detected by Ki-67 staining and flow cytometry (mean ± SEM, from 3 experiments, n = 6; **, $p < 0.01$). **(d)** Percentage of dead cells in 2D and 3D MSCs detected by propidium iodide (PI) staining (mean ± SEM, from 3 experiments, n = 6).

Figure S2

a

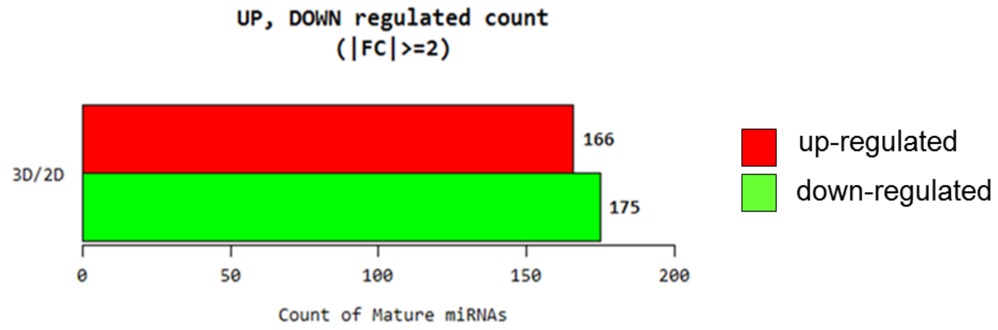

b

Up in 3D	hsa-miR-146b-5p, hsa-miR-136-5p, hsa-miR-4792, hsa-miR-340-5p, hsa-miR-101-3p, hsa-miR-4488, hsa-miR-136-3p, hsa-miR-6087, hsa-miR-379-5p, hsa-miR-889-3p, hsa-miR-337-5p, hsa-miR-193a-3p, hsa-miR-7704, hsa-miR-376c-3p, hsa-miR-7641, hsa-miR-376a-3p, hsa-miR-1185-5p, hsa-miR-124-3p, hsa-miR-34a-3p, hsa-miR-3607-3p, hsa-miR-873-5p, hsa-miR-29c-3p, hsa-miR-15a-5p, hsa-miR-5701, hsa-miR-455-5p, hsa-miR-106b-5p, hsa-miR-2355-5p, hsa-miR-146a-5p, hsa-miR-218-5p, hsa-miR-708-5p, \hsa-miR-655-3p, hsa-miR-98-5p, hsa-miR-4448, hsa-miR-10b-3p, hsa-miR-21-3p, hsa-miR-411-5p, hsa-miR-143-3p, hsa-miR-29b-3p, hsa-miR-381-3p, hsa-miR-1307-5p, hsa-miR-132-5p, hsa-miR-101-5p, hsa-miR-660-5p, hsa-miR-199a-3p, hsa-miR-199b-3p, hsa-miR-1197, hsa-miR-212-3p, hsa-miR-374a-5p, hsa-miR-382-3p, hsa-miR-378d, hsa-miR-34a-5p, hsa-miR-194-5p
Down in 3D	hsa-miR-7974, hsa-miR-503-3p, hsa-miR-145-5p, hsa-miR-193b-5p, hsa-miR-365a-5p, hsa-miR-29b-1-5p, hsa-miR-548av-3p, hsa-miR-129-2-3p, hsa-miR-548o-3p, hsa-miR-23a-5p, hsa-miR-6511a-5p, hsa-miR-335-3p, hsa-miR-6511b-5p, hsa-miR-2110, hsa-miR-4521, hsa-miR-423-5p, hsa-miR-675-5p, hsa-miR-589-3p, hsa-miR-129-1-3p, hsa-miR-1254, hsa-miR-92b-5p, hsa-miR-615-5p, hsa-miR-1268a, hsa-miR-221-5p, hsa-miR-6840-5p, hsa-miR-4326, hsa-miR-505-3p, hsa-miR-222-3p, hsa-miR-1268b, hsa-miR-6511a-3p, hsa-let-7b-3p, hsa-miR-3180<br>hsa-miR-3180-3p, hsa-miR-455-3p, hsa-miR-548ah-3p, hsa-miR-4745-5p, hsa-miR-942-5p, hsa-miR-767-5p, hsa-miR-195-3p, hsa-miR-485-5p, hsa-miR-125a-5p, hsa-miR-6735-5p, hsa-miR-1303, hsa-miR-4781-3p, hsa-miR-500a-3p, hsa-miR-6501-5p, hsa-miR-7706, hsa-miR-370-3p, hsa-miR-3187-3p, hsa-miR-128-1-5p

Figure S2. Changes in miRNA expression levels in 3D culture. (a) Differential expression of miRNAs in 2D and 3D MSCs. The total numbers of miRNAs exhibiting differential expression are shown. **(b)** Top 50 upregulated and downregulated miRNAs in 3D spheroid MSCs. Among the differentially expressed miRNAs, the top 50 miRNAs exhibiting significant changes are listed.

Figure S3

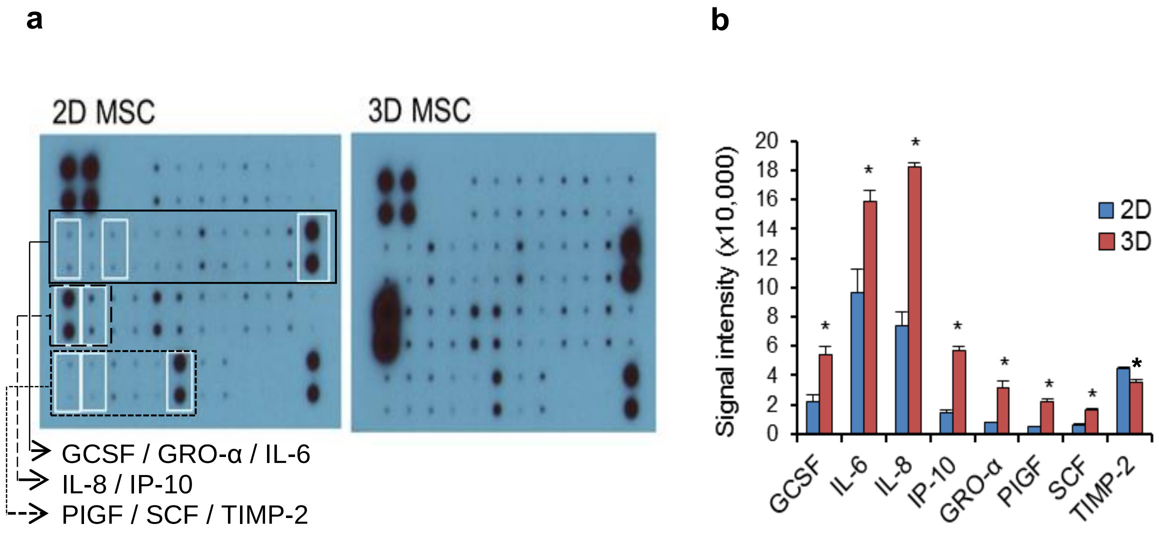

Figure S3. Cytokine secretion of 3D and 2D MSCs analyzed by membrane array. (a) Representative images of protein blots for secretary proteins in the conditioned media from 2D or 3D MSCs. Proteins showing significant changes in secretion levels are indicated with boxes. **(b)** The signal intensity of membrane spots for individual proteins from 2D or 3D MSCs were quantitatively analyzed. (mean ± SEM, from 3 experiments, n = 3; *, $p < 0.05$).

SUPPLEMENTAL TABLES

Table S1. Fold change of TGF-β related gene expressions in 3D MSC relative to 2D MSC.

Gene symbol	Description	3D/2D
**BMP2**	bone morphogenetic protein 2	**15.2**
**TGFB3**	transforming growth factor, beta 3	**13.5**
**LTBP1**	latent transforming growth factor beta binding protein 1	**3.5**
**ZFYVE16**	zinc finger, FYVE domain containing 16	**3.1**
**TGIF2**	TGFB-induced factor homeobox 2	**3.0**
**INHBA**	inhibin, beta A	**2.7**
**TGFB2**	transforming growth factor, beta 2	**2.5**
**ID4**	inhibitor of DNA binding 4, dominant negative helix-loop-helix protein	**2.3**
**DCN**	decorin	**2.3**
**NBL1**	neuroblastoma 1, DAN family BMP antagonist	**2.2**
**BMPR1B**	bone morphogenetic protein receptor, type IB	**2.1**

Table S2. **A list of top 50 transcriptomes differentially expressed in 3-D spheroid MSCs relative to 2-D cultured MSCs.**

Up in 3D	CCND3, BTBD11, ZNF154, LIF, ZNF195, BCL2L11, LONRF3, NNAT, BHLHB9, HTR7, WDR86, LTB4R2, ITGA10, ECE1, HSD11B1, PNCK, PROM2, IKBKB, ZNF302, IL7, ZNF83, EED, C3orf35, SYTL3, ZNF565, TC2N, IL18R1, NR6A1, NAPEPLD, PARD3, BARD1, TMEM175, TFPI2, CREM, WDR86, MRAS, TNIP1, CYP24A1, CPEB1, CALML4, HSD11B1, RASD1, GSN, TUBD1, C12orf56, IL33, KDM6A, UBOX5, RHD, PDE4B
Down in 3D	CARD16, MTHFSD, PHPT1, HDAC9, ANAPC15, MORF4L2, RABEP1, C1orf198, LRRC39, TRPC4, MGST1, BCAS4, KANK1, HCST, LPPR4, KIRREL3, MUTYH, GMFG, KHDC1, EPHA5, BBS9, TDRKH, MEIS2, FAM134B, BCAS4, ZNF285, INPP5F, MADD, KIAA1191, EXOSC2, TMEM9, SRP14-AS1, ZNF385B, GRAMD3, RCN2, DYSF, TP53TG3D, FNDC5, RCHY1, JADE1, LOC100268168, TRPC4, GSTT1, TPGS2, RUNX1T1, TNFAIP8, TTLL1, PDZD7, MIR143HG, ZNF823

Table S3. **List of significantly enriched gene sets (GO terms) in GSEA.**

Up-regulated in---	Gene ontology	Genes	P value	FDR	FWER
**in 3D**	CHEMOKINE_RECEPTOR_BINDING	20	0.0000	0.0003	0.0020
	CHEMOKINE_ACTIVITY	20	0.0000	0.0005	0.0010
	G_PROTEIN_COUPLED_RECEPTOR_BINDING	24	0.0000	0.0005	0.0020
	SUGAR_BINDING	18	0.0276	0.1234	0.5990
	EXTRACELLULAR_SPACE	146	0.0000	0.1400	0.7250
**in 2D**	EARLY_ENDOSOME	18	0.0000	0.0230	0.0430
	HISTONE_DEACETYLASE_COMPLEX	20	0.0000	0.0580	0.2000
	TRANSFERASE_ACTIVITY_TRANSFERRING_ALKYL_OR_ARYLOTHER_THAN_METHYLGROUPS	27	0.0011	0.0620	0.3450
	MEIOSIS_I	17	0.0012	0.0637	0.3140
	HYDROLASE_ACTIVITY_ACTING_ON_CARBON_NITROGEN_NOT_PEPTIDEBONDSIN_LINEAR_AMIDES	17	0.0025	0.0644	0.1670
	HYDROLASE_ACTIVITY_ACTING_ON_CARBON_NITROGEN_NOT_PEPTIDEBONDS	33	0.0000	0.0743	0.3060
	ENDOSOME	60	0.0000	0.0831	0.4690
	AEROBIC_RESPIRATION	15	0.0025	0.1311	0.6840
	CELLULAR_RESPIRATION	19	0.0012	0.1475	0.7500

Table S4. **List of primers used in this study.**

Gene	BP	Forward sequence	Reverse sequence
GAPDH	104	CCCCACCACACTGAATCTCC	GGTACTTTATTGATGGTACATGACAAG
OCT4	324	CGAGCAATTTGCCAAGCTCCTGAA	TTCGGGCACTGCAGGAACAAATTC
NANOG	158	CAAAGGCAAACAACCCACTT	TCTGCTGGAGGCTGAGGTAT
SOX2	126	AGCTACAGCATGATGCAGGA	GGTCATGGAGTTGTACTGCA
HMGA1	74	TCCAGGAAGGAAACCAAGG	AGGACTCCTGCGAGATGC
KLF4	105	TCTCAAGGCACACCTGCGAA	TAGTGCCTGGTCAGTTCATC
C-MYC	311	GGAGGGAGGGATCGCGCTGA	CGCGTCCTTGCTCGGGTGTT
SMARCA1	235	CCTAAACGAGAACGCAAAGC	CCAGAGCTGGATTTGGGATA
SMARCA2	165	TCATCTTTGACAGCGACTGG	TCTGATCCACGTTCAGCTTG
SMARCA4	141	AGTGCTGCTGTTCTGCCAAAT	GGCTCGTTGAAGGTTTTCAG
SMARCA5	87	AGTAACCAACAGTGGCAAAATGGTG	TGACTGAAGATTAGTACTCGTGAACC
CHD1	218	AATGGAAGTGAAGGGAGGCG	GCATCTCGAGCAATTGCATC
KMT1A	99	ATCCGCGAACAGGAATATTACC	GAGGATACGCACACACTTGAGATT
KMT1B	93	TCTATGACAACAAGGGAATCACG	GAGACACATTGCCGTATCGAG
KMT1C	57	CCGAGAGAGTTCATGGCTCTT	TGGGCAGGGTTTCTTCACTAC
KMT1E	50	TAAGACTTGGCACAAAGGCAC	CTGGCCCAACTGTCTGGATG
KMT1F	136	AGGGAGCACAAAAGGACTCA	GTGGTCGCCTGGTTACATCT
KMT2A	118	CAGCCATTTGCCTACGCTACC	TCAGTAAGAACTGGTGGATCAGG
KMT2B	117	ACCTGGGAATGACTCTAAGATGT	CACGCCTTGCACTTCCAAGA
KMT2C	62	GGACAGAGAAAAGAACGATCTCC	TGGGTGCTTACACTTACACAAGA
KMT2E	161	TTGCCCTATGCGGACCATAAT	CCATCCTCAGATGTGCTGATTG
KMT2F	77	GGCCAGATTCATCAACCACT	CGATCTTCTTCTGGGACTCG
KMT2G	108	GCTGTCGGTGCCCAAATTC	CTCAGGAAGTTTTCACGGATGTT
KMT2H	188	ACACTGTCCTTCAAAACGAGAC	GAAGAGTAGATGGCGTTGCATTA
KMT3A	57	ACAGGTTTCATCAAAGGACCAAT	GGGCAAAAATCGACTAGAAGCA
KMT3B	146	AGAAAAAGTCTACGCCACTGAAG	ATAGGGCCTCCGGTTGGAA
KMT3C	130	CTCCAAGCATCTCGGATTCCC	TGCAACATCAGGAAATATCGCTG
KMT3D	73	TCGGAGGTCCTTTCCTACCT	TATAGCCGTCCACCATCCTC
KMT3E	71	AGTTGGTGTTGGCCTATATCCC	ACACAATCGAACAGTTGGGGT
KMT3F	87	GGTGGGAACCTATCCTTGGT	CGGGCACCTCTTGTGTTAAT
KMT3G	140	GAGAGCACGCTACAACACCA	GTGTGTCCTCAGCTTCAGCA
KMT4	77	CATCCGATGGGTCTGTGA	TGGTGTCATAGTCAATTAAAACGTAATTC
KMT6A	178	AAGTACACGGGGATAGAGAATGT	GGTGGGCGGCTTTCTTTATCA
KMT6B	121	CCCTGACCTCTGTCTTACTTGTGGA	ACGGTCAGATGGTGCCAGCAATA
KDM3A	63	GTCAACTGTGAGGAGATTCCAGC	AACTTCAACATGAATCAGTGACGG
KDM3B	208	AACTTCCTCAAACCCCCTTG	CCCATCACCATCTCCTTCAC
KDM3C	205	TCCAGAATCCCAGTCACCAC	CAGCAAATCCCGTAAGGTTG
KDM4A	99	CCTTTGCTTGGCACACTGAAGACA	TTCCATGCTCAGGTGGAACAGAGT
KDM4B	95	ATCCAGAAGAAGGCCATGACAGTG	TCAAGGTCATCAAAGTCCTGGTGC
KDM4C	179	GCTCATTCCAGCACCAATTCAGCA	AGTTCTTCCAGTACTTGCGCTCCA
KDM4D	84	GGGCAACCACGATCTTTACAA	TGCAGAGGATACACCCAGTGAACA
KDM5A	111	AAGAGAAGAAGATGACGATGATGAC	CCTGAAGCCTGGTGTCTGG

KDM5B	117	ATTGCCTCAAAGGAATTTGGCAGTG	CATCACTGGCATGTTGTTCAAATTC
KDM5C	189	CATCATGGTGCAAGAAGAGC	ATGTGGGAAAGGCAGACAAG
KDM6A	247	TGAGGAGGCCTGGTACTGT	TACAAATCCGAACAACCC
KDM6B	204	CTGATGCTAAGCGGTGGAAG	TGTTGATGTTGACGGAGCAG
Snai1	135	GCGAGCTGCAGGACTCTAAT	GGACAGAGTCCCAGATGAGC
Snai2	534	ATACCACAACCAGAGATCCTCA	GACTCACTCGCCCCAAAGATG
ZEB1	237	TGCACTGAGTGTGGAAAAGC	TGGTGATGCTGAAAGAGACG
ZEB2	204	CGCTTGACATCACTGAAGGA	CTTGCCACACTCTGTGCATT
TWIST1	125	TGCCAATCAGCCACTGAAAGG	TTTGCAGGCCAGTTTGATCCC
HDAC8	78	TCCCGAGTATGTCAGTATATATGA	GCTTCAATCAAAGAATGCACCAT
HDAC9	102	TGGAGCAGCAGAGGCAAGAA	TTGCCACTGCCCTTTCTCGT
HDAC10	102	CGGCTGCTCTGGGACGACCC	CAGACACCTCTGTTCCAGGC
HDAC11	109	GGAGCTGGAGTGGGGCACAG	GCCTGCATTGTATACCACCA
NESTIN	167	GAAACAGCCATAGAGGGCAAA	TGGTTTTCCAGAGTCTTCAGTGA
PRX1	219	TCCCTCCTCAAATCCTAC	ACTATATTCCTTGGCCTTC

Table S5. **List of miRNA sequences used to induce EMT in 2D MSCs**

miRNA	Sequence
miR-146b mimic	5' : UGAGAACUGAAUUCCAUAGGCU<br>3' : UGCCCUGUGGACUCAGUUCUGG
miR-379 mimic	5' : UGGUAGACUAUGGAACGUAGG<br>3' : UAUGUAACAUGGUCCACUAACU
miR-365a-5p inhibitor	UCCCUGAAAACCCCCGUCUACAC
miR-29b-1-5p inhibitor	CGACCAAAGUAUACCACCAAAUGU
miR-129-2-3p inhibitor	UUCGGGAAUGGGGUUUUUCGUA
miR-145-5p inhibitor	CAGGUCAAAAGGGUCCUUAGGGA
miR-193b-5p inhibitor	GCCCCAAAACUCCCGCUCUACU
miR-503-3p inhibitor	CCCCAUAACAAAGGCGAACGGUCC

Full length (unedited) gels and blots

Full unedited gels for Fig. 1e

RT-PCR

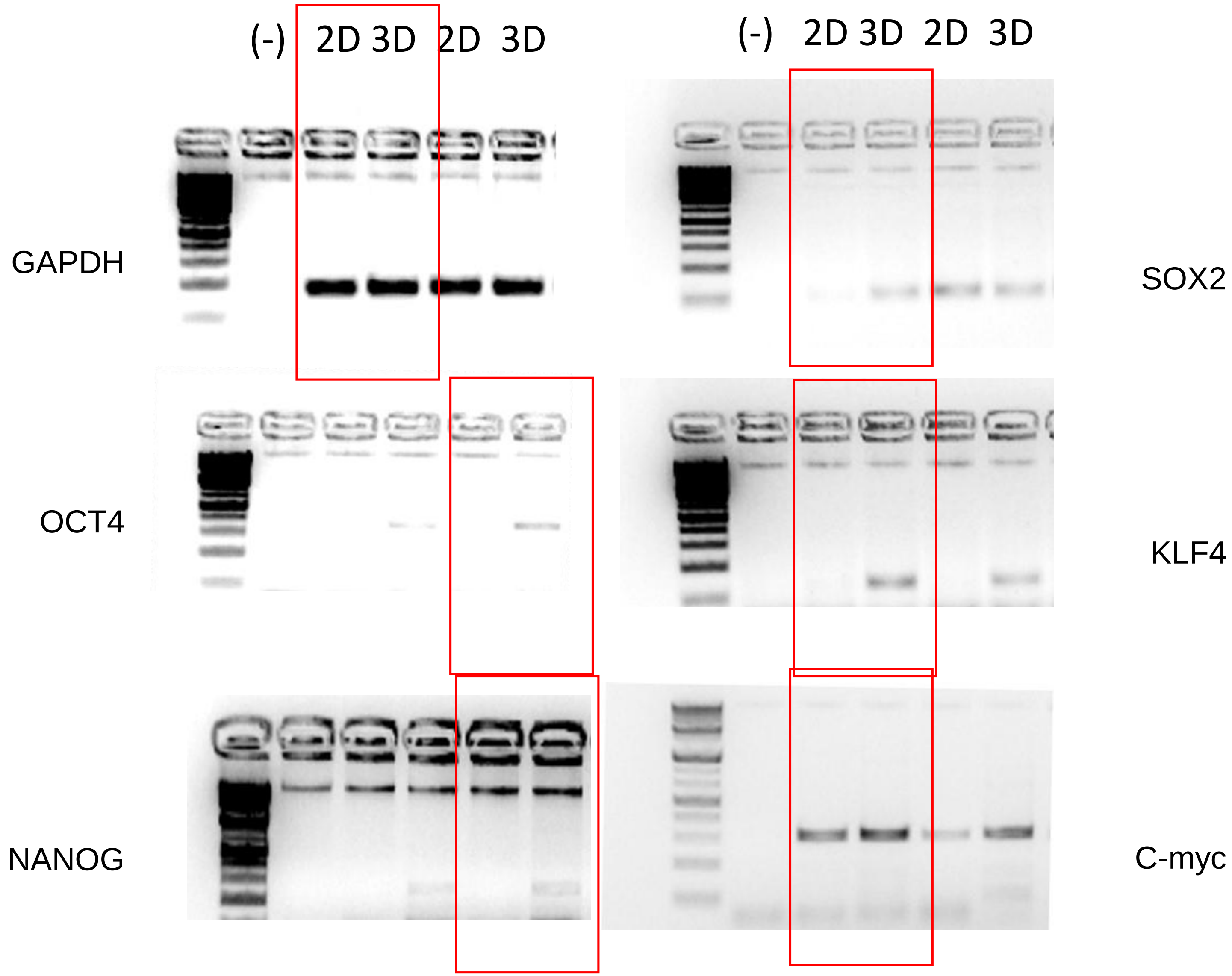

* Red lines show the lanes corresponding to those in figure

Full unedited blots for Fig. 1h

(Western blot)

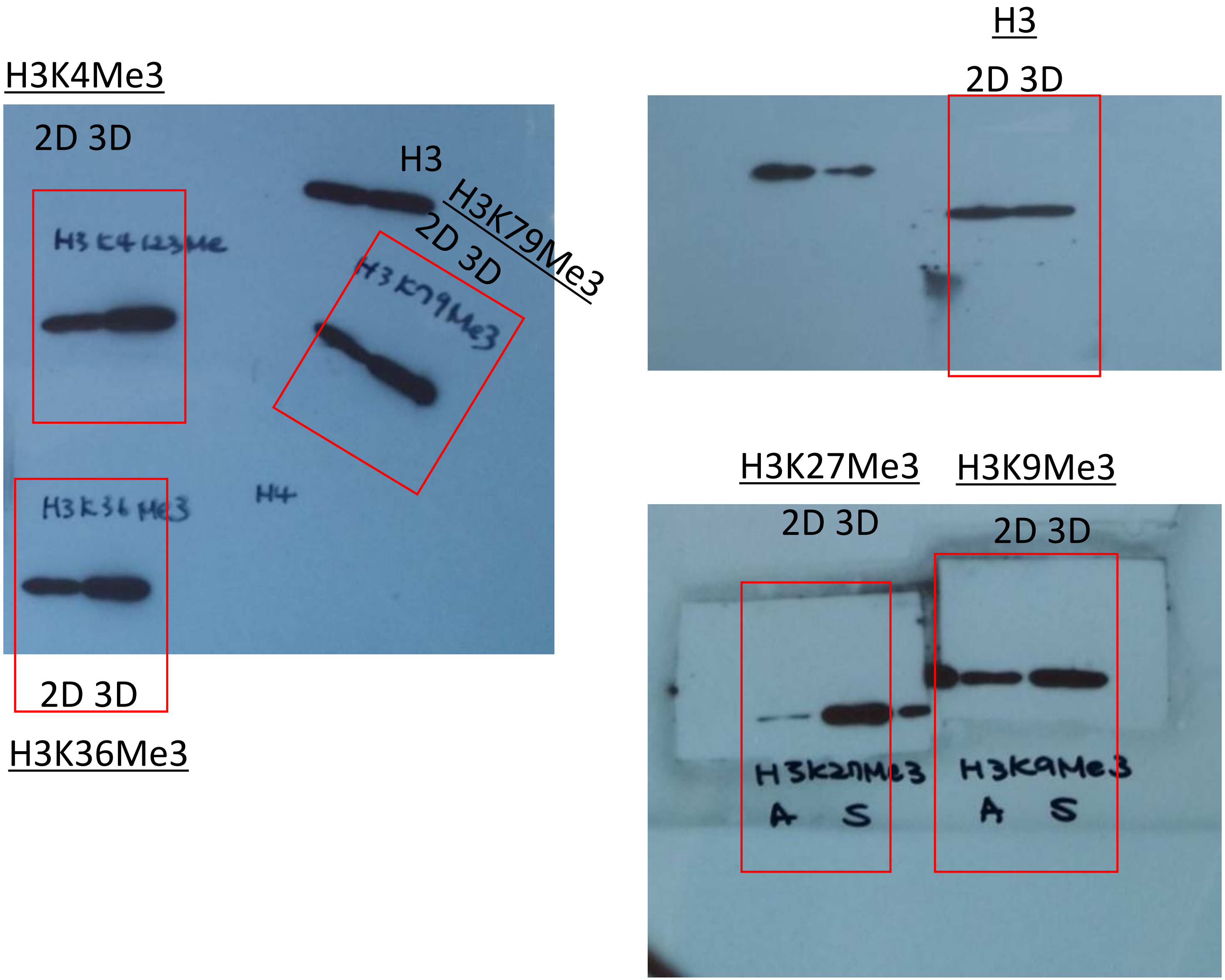

* Red lines show the lanes corresponding to those in figure
